# A Re-analysis of Our Current Understanding of Isthmus-Dependent Atrial Flutter: Some Gaps, Some Hypotheses

**Published:** 2001-10-01

**Authors:** Ashish Nabar

**Affiliations:** Department of Cardiology, Academic Hospital Maastricht, Maastricht, The Netherlands

**Keywords:** Atrial arrhythmia, macro-reentry, ablation

## Abstract

The macro-reentrant circuit of isthmus-dependent atrial flutter (AFL) is located in the right atrium around the tricuspid annulus. High acute success and low recurrence rate makes isthmus ablation a definitive therapy for patients with only AFL. However, a review of the literature suggests that, different aspects of this macro-reentrant circuit are still not entirely understood, while new information continues to emerge. The aim of this article is to discuss some gaps in our "complete" understanding of isthmus-dependent AFL. Few hypotheses have been stated which are open to investigation.

## "All scientific truth is conditioned by the state of the knowledge at the time of its announcement"

(William Osler, MD, FRCS. Harvenian Oration, Royal College of Physicians, 1906.)

A recent population-based US study suggested that the incidence of atrial flutter (AFL) ranged from 5/100,000 in those < 50 years old to 587/100,000 in subjects older than 80 [1]. Further, men and individuals with pre-existing heart failure or chronic obstructive pulmonary disease were at highest risk. Although the combination of multiple electrical cardioversion and serial anti-arrhythmic drug (AAD) therapy is effective in maintaining sinus rhythm for a long time [2],  acute success (> 90%) and a low recurrence rate (< 10%) of radiofrequency ablation (RFA) makes the latter a compelling choice [3,4].  Intuitively therefore, it seems no longer necessary to dwell on the mechanisms of isthmus-dependent AFL. A review of the literature, however, suggests that, different aspects of the macro-reentrant circuit of this potentially curable atrial arrhythmia are not entirely understood, while new information continues to emerge. The aim of this article is to discuss some gaps in our "complete" understanding of isthmus-dependent AFL. Few hypotheses have been stated which are open to investigation.

## How well do we understand the re-entrant right atrial (RA) activation during isthmus-dependent AFL?

The question is, does a large RA circuit such as typical AFL require a specific zone of slow conduction so that the circuit length (wavelength, a product of refractory period and conduction velocity [5]) can fit the anatomical pathway? Lately, different groups have mapped the RA circuit of human AFL in great detail using multiple endocardial recordings [6,7], intra-cardiac echocardiography [8],  electro-anatomical CARTO [9], 64-electrode basket catheter [10], non-contact EnSite [11] and body surface mapping [12].  There is a general agreement between these studies and other protocols [13,14], measuring conduction velocity directly, regarding slow isthmus conduction during AFL. This finding has been substantiated using unipolar electrograms [15].   The criss-cross muscular trabecular pattern in the isthmus seems to favor non-uniform anisotropic conduction [16]. Kinder et al., then seem to be the only group disputing the presence of slow conduction in this region [17]. 

Recently, using the CARTO system, we re-constructed a bi-atrial map of isthmus-dependent AFL [18].  The right and the left side of the atrial septum was mapped separately. The initiation of left atrial (LA) activation from the RA flutter circuit by a discrete septal breakthrough, sometimes more than one, supported that the inter-atrial conduction is via specific, functionally active trans-septal pathway(s), corresponding to the sites previously identified: inferior coronary sinus - LA connection, Bachmann's bundle and the fossa ovalis [19]. Interestingly, the sequence of activation on the right and the left side of the septum was not always synchronous. For example, we observed in a patient with clockwise (CW)-AFL that the right septum was activated in a supero-inferior direction but the spread of activation in the left septum, following a breakthrough in the region of fossa ovalis, was supero-inferiorly as well as infero-superiorly. Does this argue for an electrically separate behaviour of the right and the left atrial septum?

Is the upper turn around site of the RA flutter circuit, anterior or posterior to the superior vena cava (SVC)? Detailed mapping has shown that activation often proceeded on both sides of the SVC [9,18]. Which limb of activation is then a part of the macro-reentrant circuit? Entrainment studies suggest activation anterior to the SVC orifice, between the SVC and tricuspid annulus, as the upper turn around site of the flutter circuit [6,20]. This "superior isthmus" is, however, too wide to be a routine target for flutter ablation.

Until recently it was unanimously believed that the crista terminalis formed the posterior boundary of the AFL circuit. The study of Friedman et al., brought new information, suggesting that the postero-medial RA (sinus venosa region) provided the posterior functional line of block [7]. This was based on their observation that, during counterclockwise (CCW) and CW-AFL, the posterior RA wall activation was consistent from patient to patient, along the direction of activation of anterior RA wall, but opposite to the direction of septal activation. Furthermore, they reported double potentials in the postero-medial RA region, suggesting a turn around point. Although their reasoning is convincing, we [18] as well as Shah et al., [9] have found heterogeneity in the activation of posterior RA wall.

## Is crista terminalis, in patients with AFL, always impermeable? Clinical significance

The crista terminalis is a thick muscular ridge beginning at the superior septal surface, running anterior to the SVC orifice, down the anterior RA wall, and then proceeding between the tricuspid annulus and the inferior vena cava (IVC). It ends as Eustachian valve. Poor transverse conduction across the crista is important to hold the flutter pathway against the tricuspid annulus, in other words, to maintain the posterior boundary of the flutter circuit [21,22]. Some findings discussed below suggest that transverse coupling may occur at various sites along the crista.

Recently, the group of Scheinman described atypical forms of RA-AFL [23]. These are generally faster than the associated typical AFL and often transient. Lower-loop re-entry is an isthmus-dependent AFL characterized by re-entry around the IVC, following a breakthrough low or high in the crista terminalis. Also, multiple cristal breakthroughs were observed during acceleration of some episodes of typical AFL. A break down of the posterior functional crista barrier has been suggested as one of the mechanism of transition from AFL to atrial fibrillation (AF) [22,24].

While judging the success of AFL ablation, both bi-directional isthmus conduction (BIC) block and a complete isthmus-line of double potentials are complementary to each other [25]. The routine demonstration of BIC and block are based on the assumption of lack of transverse conduction across crista terminalis. Occasionally, during coronary sinus (CS) pacing after isthmus ablation, despite the presence of complete isthmus line of double potentials, the activation pattern recorded by the Halo catheter does not show an isthmus conduction block. This could be confusing and may lead to unnecessary additional radiofrequency pulses. A study by Scaglione et al., explained that the apparent conduction over the isthmus during pacing from proximal CS was due to conduction along the posterior part of the IVC orifice, which then activated the low anterior RA via a cristal breakthrough [26].

## Do we understand completely the electrocardiographic inscription of CCW- and CW-AFL?

Lai et al., dismissed the role of flutter wave polarity to distinguish between CCW and CW rotation of AFL [27]. Neither was the 3-dimensional CARTO mapping of the RA helpful [9]. Bystander atrial regions, posterior RA and LA, might be important contributors to the ECG flutter wave morphology. Okumura et al. [28], and Schoels et al. [29], using animal models of AFL, have since long suggested a role of LA activation.

An infero-superior septal activation, after the exit of the flutter wave from the RA isthmus, and a supero-inferior anterior RA wall activation, following thereafter, is the principal generator of the basic - initial negative followed by terminal positive - inferior ECG-lead deflection of CCW-AFL [10,18]. Frequently, variations are observed in the extent of initial negative polarity and the terminal positive part of the flutter wave [30]. In our study, patients with CCW-AFL showed consistent infero-superior LA activation, starting soon after isthmus activation,  and therefore synchronous with septal activation. The total LA activation time was always shorter, and as a proportion of AFL cycle length varied from patient to patient. Consequently, the overlap of LA activation with the oppositely directed supero-inferior anterior RA wall activation showed inter-individual variations. Summarily, the LA activation definitely influences the initial negative part of the flutter wave, but the extent to which it modifies the terminal positive deflection varies from patient to patient.

CW-AFL is generally recognized to have a small initial negative followed by a prominent and notched positive flutter wave in the inferior leads. This morphology correlates well with an initial infero-superior anterior RA wall activation followed by a supero-inferior septal activation. The positive part of the flutter wave after the notch is believed to result from a supero-inferior LA activation via the Bachmann's bundle. Controversially, Saoudi et al., reported the classical inferior-lead pattern - prominent negative followed by small terminal positive flutter waves - in a majority of their patients with CW-AFL [31]. Our study could not corroborate that LA activation during CW-AFL was always via Bachmann's bundle and therefore responsible for the positive part of the flutter wave after the notch [18]. Because of the infrequency of CW-AFL, <10% of CCW-AFL population, only a small number of patients have been studied by each of the previous authors. Variations reported in the ECG and the bi-atrial activation pattern of CW-AFL make it difficult to arrive at a consensus.

## Isthmus-dependent AFL associated with other heart disease. Have we acknowledged enough?

Lately there has been a focus on macro-reentrant atrial tachycardias after cardiac surgery. Some reports suggest that 40%-70% of intra-atrial re-entrant circuits after Mustard, Senning or Fontan operation, or after complete repair of atrial septal defect, tetralogy of Fallot, or a Rastelli procedure are isthmus-dependent [32,33]. In the remaining cases, the re-entrant circuits were associated with lateral right atriotomy scar or atrial septal patch. Akar et al., found that multiple circuits, including isthmus-dependent AFL, could coexist and surface ECG morphology does not provide adequate discrimination [34]. Isthmus-dependent AFL has been seen after mitral valve replacement [32]. and in the donor atrium after orthotopic heart transplant [35]. Hypertrophic [36]. and tachycardia-induced [37]. cardiomyopathy are also associated with isthmus-dependent AFL. CARTO mapping is certainly useful in post-surgery atrial arrhythmias. It helps to distinguish a focal from a re-entrant mechanism. Moreover, the unidentified circuit path can be spatially reconstructed. We studied 15 patients with AFL after repair of atrial septal defect or Ebstein's anomaly and replacement/repair of mitral or aortic valve. Electrocardiographically, AFL was typical, with negative (n = 11) or positive (n = 2) inferior-lead flutter waves in a majority of patients, and atypical in 2. During electrophysiological study AFL was documented in 13 patients, and CCW rotation (n = 11) was frequently induced. Isthmus ablation was successful in 13 (87%) patients.

In the present-days it is important to recognize AFL in the setting of an implanted pacing or a shocking device. Physiological pacing does not prevent recurrence of AFL, therefore ablation should be considered when AFL is detected [38]. In patients with an implantable atrial defibrillator [39]. better algorithms are required to distinguish AFL from AF, because some devices (7250 Jewel® AF Arrhythmia Management Device, Medtronic, Inc) can over-pace AFL. Ablation could be considered to reduce the number of device shocks. Even with a dual-chamber ICD, AFL with fast ventricular rate could lead to inappropriate shocks [40]. A recent study showed that, none of the patients after isthmus ablation had inappropriate ICD therapies for AFL [41].

## "Organized AF". Could it be sometimes isthmus-dependent?

About 10-30% of patients with AFL have associated AF [38,42]. Further, compared to patients without treatment or those treated with rate-control therapy, use of AADs for prophylaxis of AF is associated with a three-fold increased probability of AFL recurrence [43]. Current literature supports that isthmus ablation for AFL could reduce recurrences of associated AF [4,44]. Since the first publication by Huang et al., there has been more evidence that RFA of "AAD-induced AFL" could palliate recurrent AF [4,45,46].

We need to discuss an electrophysiological entity encountered often in the laboratory: ECG shows coarse AF. A Halo catheter, positioned around the tricuspid annulus, shows a repetitive CCW activation pattern with beat-to-beat variations in the cycle length (170-210 ms). Electrograms obtained from the coronary sinus may show 1:1 RA:LA response or disorganized atrial activity. What are we dealing with? Is it "organized AF"? According to one study, the cycle length variability of AFL should not exceed 11.5 ms and the index of irregularity (ratio of mean cycle length and standard deviation of mean cycle length) should be < 7.5% [47].

Our experience after isthmus ablation in 3 patients with similar electrophysiological features was dismal [4]. Coarse AF did not stop and all patients continued to have recurrences. However Kumagai et al., after isthmus ablation in a similar group of patients (n = 16) were successful in preventing AF in 75% patients at 1-year follow-up [48]. Higher F wave amplitude in lead V1, left ventricular ejection fraction and left atrial dimension were significant predictors of success.

The favorable results of Kumagai et al., raises mechanistic issues. Is it likely that, in some patients this particular type of AF is associated with a large "mother wave" in the RA and small dependent "daughter waves" in LA? [49]. A repetitive CCW activation pattern along the Halo catheter suggests a constant trajectory along the anterior RA wall and through the isthmus. Beat-to-beat variations in the remaining re-entrant trajectory, probably RA septum and roof, may then be responsible for the observed cycle length variations of this arrhythmia, which is otherwise "organized". Conduction to LA may be 1:1 or fibrillatory. A high-density mapping system, such as the non-contact EnSite, could be used to obtain a beat-to-beat map of this arrhythmia, to determine how often the re-entrant pathway traverses the isthmus. Could this exercise predict patients who will benefit from isthmus ablation? Interruption of the leading RA "mother wave" may render the remaining LA wavelets unsustainable. This proposal is based on the current understanding of the re-entrant nature of AFL and AF, but is hypothetical and certainly open to investigation.

## Figures and Tables

**Figure 1:**
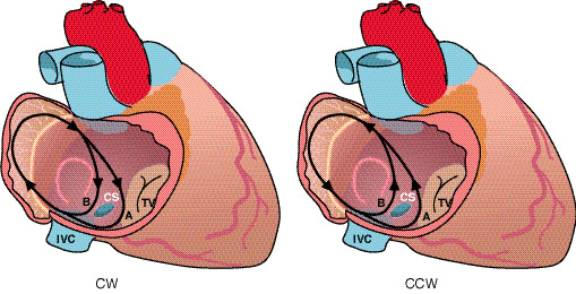
A schema of the macro-reentrant circuit of isthmus-dependent atrial flutter (AFL) in right atrium (RA). In either rotation, counterclockwise (CCW) or clockwise (CW), the activation wave front could propagate either anterior (A) or posterior (B) to the coronary sinus (CS). IVC, inferior vena cava; TV, tricuspid valve

**Figure 2:**
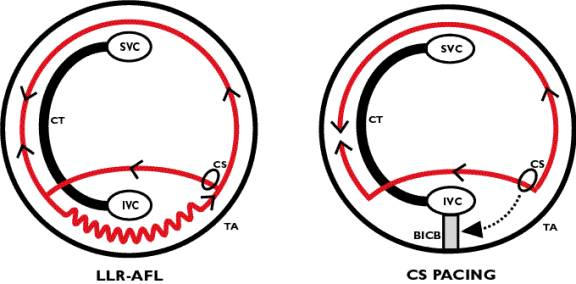
The panel on the left shows the location of the lower-loop AFL re-entrant circuit (LLR-AFL) round the IVC. A Halo catheter placed around the tricuspid annulus (TA) would show a collision of two wave fronts along the anterior RA wall. The panel on the right explains why after isthmus ablation, despite a complete line of double potentials, conduction over the isthmus could be thought to be seemingly present. During CS pacing, conduction is along the posterior part of IVC orifice, which then activates the lower anterior RA via crista terminalis (CT) breakthrough.

**Figure 3:**
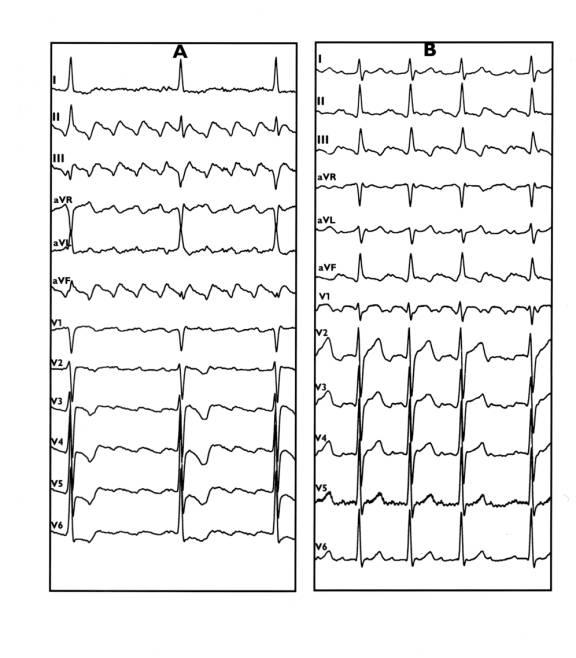
12-lead ECG of CCW (A) and CW (B) AFL.

**Figure 4:**
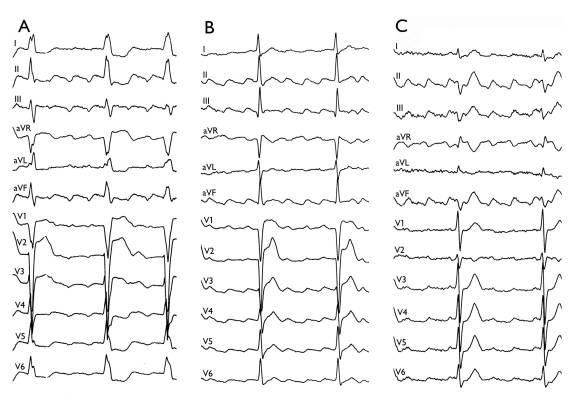
Panels A-C show 12-lead ECGs of 3 patients with CCW-AFL. A general, initial negative followed by a terminal positive pattern of flutter waves (FW) is observed in the inferior leads. Note the differences in the relative extent of initial negative and terminal positive part of the FWs. A) prominent negative FWs, B) equally prominent negative and positive component of the FWs, and C) a prominent terminal positive component of the FWs

**Figure 5:**
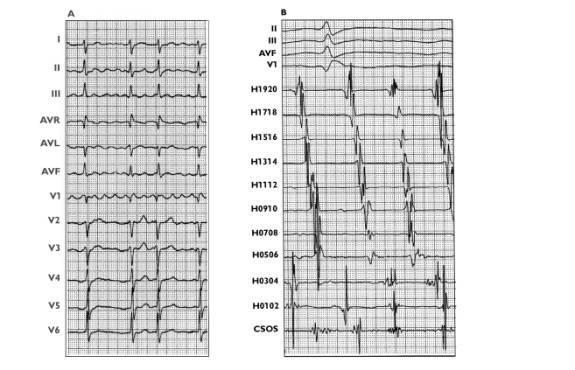
Panel A shows coarse AF. Panel B shows intracardiac electrograms from a Halo catheter positioned around the TA and the CS. Note that the activation sequence in the Halo catheter is directionally stable (CCW) despite variations in cycle length. Electrograms from the CS suggest a 1:1 left atrial response.
